# Discriminative analysis of schizophrenia patients using graph convolutional networks: A combined multimodal MRI and connectomics analysis

**DOI:** 10.3389/fnins.2023.1140801

**Published:** 2023-03-30

**Authors:** Xiaoyi Chen, Pengfei Ke, Yuanyuan Huang, Jing Zhou, Hehua Li, Runlin Peng, Jiayuan Huang, Liqin Liang, Guolin Ma, Xiaobo Li, Yuping Ning, Fengchun Wu, Kai Wu

**Affiliations:** ^1^Department of Biomedical Engineering, School of Biomedical Sciences and Engineering, South China University of Technology, Guangzhou International Campus, Guangzhou, China; ^2^Department of Emotional Disorders, The Affiliated Brain Hospital of Guangzhou Medical University, Guangzhou, China; ^3^Guangdong Engineering Technology Research Center for Translational Medicine of Mental Disorders, Guangzhou, China; ^4^School of Material Science and Engineering, South China University of Technology, Guangzhou, China; ^5^National Engineering Research Center for Tissue Restoration and Reconstruction, South China University of Technology, Guangzhou, China; ^6^Guangdong Province Key Laboratory of Biomedical Engineering, South China University of Technology, Guangzhou, China; ^7^Department of Radiology, China-Japan Friendship Hospital, Beijing, China; ^8^Department of Biomedical Engineering, New Jersey Institute of Technology, Newark, NJ, United States; ^9^Department of Psychosomatic, The Affiliated Brain Hospital of Guangzhou Medical University, Guangzhou, China; ^10^Department of Psychiatry, The Affiliated Brain Hospital of Guangzhou Medical University, Guangzhou, China; ^11^Department of Nuclear Medicine and Radiology, Institute of Development, Aging and Cancer, Tohoku University, Sendai, Japan

**Keywords:** schizophrenia, graph convolutional network, discriminative analysis, human brain connectomics, multimodal MRI

## Abstract

**Introduction:**

Recent studies in human brain connectomics with multimodal magnetic resonance imaging (MRI) data have widely reported abnormalities in brain structure, function and connectivity associated with schizophrenia (SZ). However, most previous discriminative studies of SZ patients were based on MRI features of brain regions, ignoring the complex relationships within brain networks.

**Methods:**

We applied a graph convolutional network (GCN) to discriminating SZ patients using the features of brain region and connectivity derived from a combined multimodal MRI and connectomics analysis. Structural magnetic resonance imaging (sMRI) and resting-state functional magnetic resonance imaging (rs-fMRI) data were acquired from 140 SZ patients and 205 normal controls. Eighteen types of brain graphs were constructed for each subject using 3 types of node features, 3 types of edge features, and 2 brain atlases. We investigated the performance of 18 brain graphs and used the TopK pooling layers to highlight salient brain regions (nodes in the graph).

**Results:**

The GCN model, which used functional connectivity as edge features and multimodal features (sMRI + fMRI) of brain regions as node features, obtained the highest average accuracy of 95.8%, and outperformed other existing classification studies in SZ patients. In the explainability analysis, we reported that the top 10 salient brain regions, predominantly distributed in the prefrontal and occipital cortices, were mainly involved in the systems of emotion and visual processing.

**Discussion:**

Our findings demonstrated that GCN with a combined multimodal MRI and connectomics analysis can effectively improve the classification of SZ at an individual level, indicating a promising direction for the diagnosis of SZ patients. The code is available at https://github.com/CXY-scut/GCN-SZ.git.

## 1. Introduction

Schizophrenia (SZ), a severe and disabling psychiatric disease with visual and auditory hallucinations along with disorganized speech and thoughts as the common symptoms, has been a key focus of neuroimaging research for decades (Rossler et al., 2005; Tost and Meyer-Lindenberg, 2012). The diagnosis of SZ patients solely based on clinical observation may lack objectivity and accuracy because of the heterogeneous and complex clinical characteristics (Tost and Meyer-Lindenberg, 2012). Magnetic resonance imaging (MRI), as an exciting noninvasive tool to study the brain, helps to model the brain functional and structural disease mechanisms of SZ (van den Heuvel and Fornito, 2014; Kong et al., 2021). Importantly, advances in network science and graph theory have improved our ability to study the topological organization between brain regions. Brain connectivity can be measured to generate brain “connectomics” (Farras-Permanyer et al., 2015), which have been used for quantitatively analyzing regional and global network topology of the human brain (van den Heuvel and Fornito, 2014; Jiang et al., 2020; Jo et al., 2020). Abnormalities in brain structure, function and connectivity have been widely reported in SZ patients using multimodal MRI and connectomics with structural magnetic resonance imaging (sMRI) and resting-state functional magnetic resonance imaging (rs-fMRI) data. With regard to structural brain abnormalities, SZ patients have widespread cortical thinning, a smaller cortical surface, reduced gray matter volume (GMV), and reduced white matter volume (WMV), with the largest effects observed in frontal and temporal lobe regions (van Erp et al., 2018; Wu et al., 2018; Keshavan et al., 2020). Functional brain abnormalities include significantly increased regional homogeneity (ReHo) in the striatum, the right parahippocampal gyrus, and the right middle temporal gyrus (Wu et al., 2018; Li et al., 2020), significantly increased amplitude of low frequency fluctuation (ALFF) in the right fusiform gyrus and the left superior temporal gyrus (Wu et al., 2018; Li et al., 2020), and significantly decreased degree centrality (DC) in the right supramarginal gyrus, the right transverse temporal gyrus and the bilateral putamen (Chen et al., 2015; Jo et al., 2020). The human brain is a highly interconnected network, and evidence for structural and functional abnormalities in SZ patients has developed into a dysconnectivity hypothesis (Friston and Frith, 1995). More direct evidence for the dysconnectivity hypothesis comes mainly from multimodal MRI studies, which have shown widespread structural and functional dysconnectivity in brain networks in SZ (van den Heuvel and Fornito, 2014; Northoff and Duncan, 2016; Rolls et al., 2020).

In recent decades, classification studies of SZ patients have employed machine learning techniques that enable statistical inferences at the level of the individual patient (Arbabshirani et al., 2017). Deep learning (Hatcher and Yu, 2018; Le et al., 2020), as a subfield of machine learning, can create a fully automated diagnostic process with no expert clinical intervention (Qureshi et al., 2019) because of its powerful feature representation capability. However, most machine learning methods adopted in previous studies were typically based on independent neuroimaging features or connection features instead of the connectome itself (Lei et al., 2022). Currently, graphs are the most commonly used representation of brain networks in neuropsychiatric disorder diagnosis. The use of graphs provides an alternative approach to capture topological information within brain networks. Network embedding (Grover and Leskovec, 2016; Jiang et al., 2020) approach is used to transforms the nodes of a network into a lower-dimensional representation with the network structure information. Graph convolutional networks (GCNs), proposed by Kipf and Welling (2017), was a graph embedding model that effectively combined node features with structure information during the learning process. Lee et al. (2019) proposed a GCN model with a self-attention graph pooling method that achieved superior graph classification performance. Lei et al. (2022) used GCN to investigate topological abnormalities of functional brain networks in SZ and achieved a higher classification accuracy (85.8%) compared with support vector machine (SVM) (80.9%). Oh et al. (2022) developed the BrainNet-Global Covariance Pooling-Attention Convolutional Neural Network (BrainNet-GA CNN), which showed an accuracy of 83.13%. In addition to use MRI data for diagnosis of SZ patients, some studies used Electroencephalography (EEG) data. Compared to MRI data, EEG data has a comparatively cost and good temporal resolution, and therefore it is possible for studies used large data sets (Alves et al., 2022). Alves et al. (2022) built cortical networks as the input of a tuned convolutional neural network (CNN) for SZ diagnosis, and the classification performance was significantly better than using network measures to describe the network structure. Chang et al. (2021) applied GCN to mismatch negativity (MMN) brain functional networks that based on EEG data and achieved an accuracy of 93.33%, which significantly outperformed the SVM classifier trained on graph-theoretic features.

In our previous research, we proposed an integrated analysis of functional MRI and connectomics that considered characteristics of brain regions of interest (ROIs) and the functional connectivity between ROIs (Chen et al., 2023). Specifically, we obtained an average accuracy of 92.7% based on the GCN method, which outperformed the methods that focused on features of single ROIs and the methods that only based on connectomics analysis. And the GCN method performed better than the traditional machine learning method (SVM, RF: random forest, LR: logistic regression, LDA: linear discriminant analysis and KNN: K-nearest neighbor) and the traditional deep learning method (MLP: multi-layered perceptron and CNN). The results demonstrated that taking topological relationships between ROIs into account in a combined functional MRI and connectomics analysis could effectively improve the classification performance of SZ patients. However, compared to single-modal MRI data analyses using the proposed method, multimodal MRI analyses may offer better diagnosis and prediction in SZ. Therefore, in this study, we would like to investigate multimodal MRI data based on the proposed integrated analysis method, aiming to further improve the classification performance of SZ patients. In addition, different brain parcellation schemes may have an impact on the performance of classification. To verify the robustness of the proposed method, we would like to investigate the effect of different brain atlases based on the proposed method.

In this study, we applied a GCN method to the classification of SZ patients with a combined multimodal MRI and connectomics analysis. In addition, we investigated the effects of 2 brain atlases to verify the robustness of the proposed method. Furthermore, motived by the need for explainability (Li X. et al., 2021), the GCN framework contained node-selection pooling layers, which highlight salient brain regions (salient nodes in the graph) to infer the important brain regions for prediction.

## 2. Materials and methods

### 2.1. Subjects

A total of 345 Chinese Han subjects were recruited from the Affiliated Brain Hospital of Guangzhou Medical University and the local community, including 140 SZ patients and 205 NCs (Table 1). And 140 SZ patients included 61 first-episode medication-naïve SZ (FESZ) patients and 71 medicated chronic SZ (CSZ) patients. The age of the subjects was between 18–60 years and the inclusion and exclusion criteria of subjects were the same as those in our previous studies (Wu et al., 2018; Kong et al., 2021; Huang et al., 2022; Chen et al., 2023). The SZ patients were diagnosed by veteran psychiatrists according to the structured clinical interview complying with the criteria of the Diagnostic and Statistical Manual of Mental Disorders-IV-Text Revision (DSM-IV-TR) (SCID). The Positive and Negative Syndrome Scale (PANSS) scores were over 51 and 60 for FESZ patients and CSZ patients, respectively. At least three positive symptom items with a score of at least 4 were included. In addition, the FESZ patients were recruited when they were seeking help psychotic symptoms for the first time and did not take any antipsychotic medication. And the CSZ patients were all taking antipsychotic medication and the course of disease were more than 2 years. The NCs were recruited from the local community through advertisements and group matching on demographic parameters. This study was conducted following the Declaration of Helsinki and approved by the Ethics Committee of the Affiliated Brain Hospital of Guangzhou Medical University. Each subject or their legal guardian was fully aware of the details of the experiment and signed informed consent forms before enrollment.

**Table 1:** The demographics and clinical variables of the subjects.

	NC (*n* = 205)	SZ (*n* = 140)	T(χ^2^)	*p*
Sex(Male/Female)	110/95	95/45	6.378	0.012
Age	32.51 ± 8.39	34.22 ± 8.23	−1.870	0.062
Years of Education	12.84 ± 2.83	10.69 ± 3.32	6.260	< 0.001
PANSS-PScore	–	23.14 ± 5.25	–	–
PANSS-NScore	–	22.53 ± 7.48	–	–
PANSS-GScore	–	39.88 ± 9.59	–	–
PANSS-TScore	–	85.55 ± 18.55	–	–

The values are denoted as the “mean ± standard deviation.” The comparison of sex distribution was performed using the χ2 test. The comparison of age and years of education was performed using a two-sample T test. PScore: positive score; NScore: negative score; GScore: general score; TScore: total score.

The exclusion criteria for all subjects included: (1) any other psychiatric Axis I disorder that meets DSM-IV criteria, including schizoaffective disorders, intellectual disability, major depressive disorder, bipolar disorder, delirium, dementia, memory disorder, and other cognitive disorders, (2) mental disorders due to drug dependence, severe unstable somatic disease, heart disease, hypertension, definite diabetes, or thyroid diseases, (3) narrow-angle glaucoma, (4) a history of epilepsy, except for febrile convulsions, (5) alcohol dependence meeting DSM-IV-TR criteria (excluding nicotine dependence), (6) having received electroconvulsive therapy in the past 6 months, (7) any contraindications to MRI, (8) medical resource neuroleptic malignant syndrome or serious tardive dyskinesia, (9) a serious suicide attempt or an irritative state, (10) noncompliant drug administration or a lack of legal guardians, (11) lactating, pregnant, or planning to become pregnant, and (12) the NCs who had a first-or second-degree relative with a psychiatric Axis I disorder according to the DSM-IV criteria. This study was conducted following the Declaration of Helsinki and approved by the Ethics Committee of the Affiliated Brain Hospital of Guangzhou Medical University. Each subject or their legal guardian was fully aware of the details of the experiment and signed informed consent before enrollment.

### 2.2. Multimodal MRI data acquisition and preprocessing

All MRI images were acquired using a 3.0-T Philips MR Scanner (Philips, Achieva, the Netherlands) at the Affiliated Brain Hospital of Guangzhou Medical University. During the scanning process, the subjects were instructed to rest quietly in the instrument, breathe smoothly, and keep their eyes closed but not to fall asleep. For each subject, the T1-weighted sMRI images were acquired using a magnetic preparation fast gradient-echo (MPRAGE) sequence (matrix = 256 × 256 × 188; spatial resolution = 1 × 1 × 1 mm^3^; echo time (TE) = 3.7 ms, repetition time (TR) = 8.2 ms, flip angle (FA) = 7°, field of view (FOV) = 256 × 256 mm^2^, slice thickness = 1.0 mm, and slice number = 224). The rs-fMRI images were collected using an echo-planar imaging (EPI) sequence (matrix = 64 × 64 × 36; spatial resolution = 3.4 × 3.4 × 4 mm^3^; TE = 30 ms; acquisition time = 2000 ms; FA = 90°; FOV = 211 × 211 mm^2^; slice thickness = 4.0 mm, and slice number = 36).

The sMRI images were preprocessed using the SPM8 software package (http://www.fil.ion.ucl.ac.uk/spm; Institute of Neurology, University College London, London, United Kingdom). Each sMRI image was segmented into three tissue maps, including gray matter (GM), white matter (WM), and cerebrospinal fluid (CSF). The rs-fMRI images were preprocessed using SPM8 (https://www.fil.ion.ucl.ac.uk/spm; Institute of Neurology, University College London) and Data Processing & Analysis for Brain Imaging (DPABI; Yan et al., 2016). The preprocessing steps of sMRI images and rs-fMRI images were the same as those in our previous studies (Wu et al., 2018; Kong et al., 2021; Huang et al., 2022; Wang et al., 2022).

### 2.3. Brain graphs construction

#### 2.3.1. Node features extraction

Due to the different brain morphologies of subjects, a brain atlas was proposed to register the brain morphology to the same standard space. In this study, we used 2 brain atlases to divide the ROIs, including the automated anatomical labeling (AAL) atlas (Tzourio-Mazoyer et al., 2002) with 90 ROIs and the Human Brainnetome Atlas (BNA; Fan et al., 2016) with 246 ROIs. To create a brain graph, nodes were defined as ROIs, and the corresponding ROI features were defined as node features. Three sMRI measurements, including GMV, WMV and structural degree centrality (sDC), and three fMRI measurements, including ReHo, ALFF and functional degree centrality (fDC), were calculated in each ROI of the AAL and BNA atlases. The calculation methods of the ROI features used in this study were the same as those in our previous studies (Wu et al., 2018; Zang et al., 2021; Huang et al., 2022; Wang et al., 2022).

The GMV or WMV of each ROI was calculated as the average GMV or WMV of all voxels in that ROI. The ALFF method was used to measure regional spontaneous neuronal activity (Zang et al., 2007). The time series of each voxel was transformed to the frequency domain with a fast Fourier transform (FFT), and the power spectrum was obtained. ALFF was calculated as the averaged square root across 0.01–0.08 Hz and then divided by the global mean ALFF for each subject for standardization. The ALFF of a ROI was represented by the average ALFF of all voxels in the ROI. The ReHo method was used to measures the functional synchronization of a voxel with its nearest neighbors (Zang et al., 2004). The ReHo of each voxel was divided by the global mean ReHo, and the ReHo of a ROI was defined in the same way as ALFF analyses. The DC method was used to measure the importance/centrality of a node through the strength of connections to all other nodes (Wang et al., 2015). In this study, the sDC or fDC of a given ROI was defined as the sum of its structural or functional connectivity with all other ROIs.

#### 2.3.2. Edge features construction

**Figure 1:**
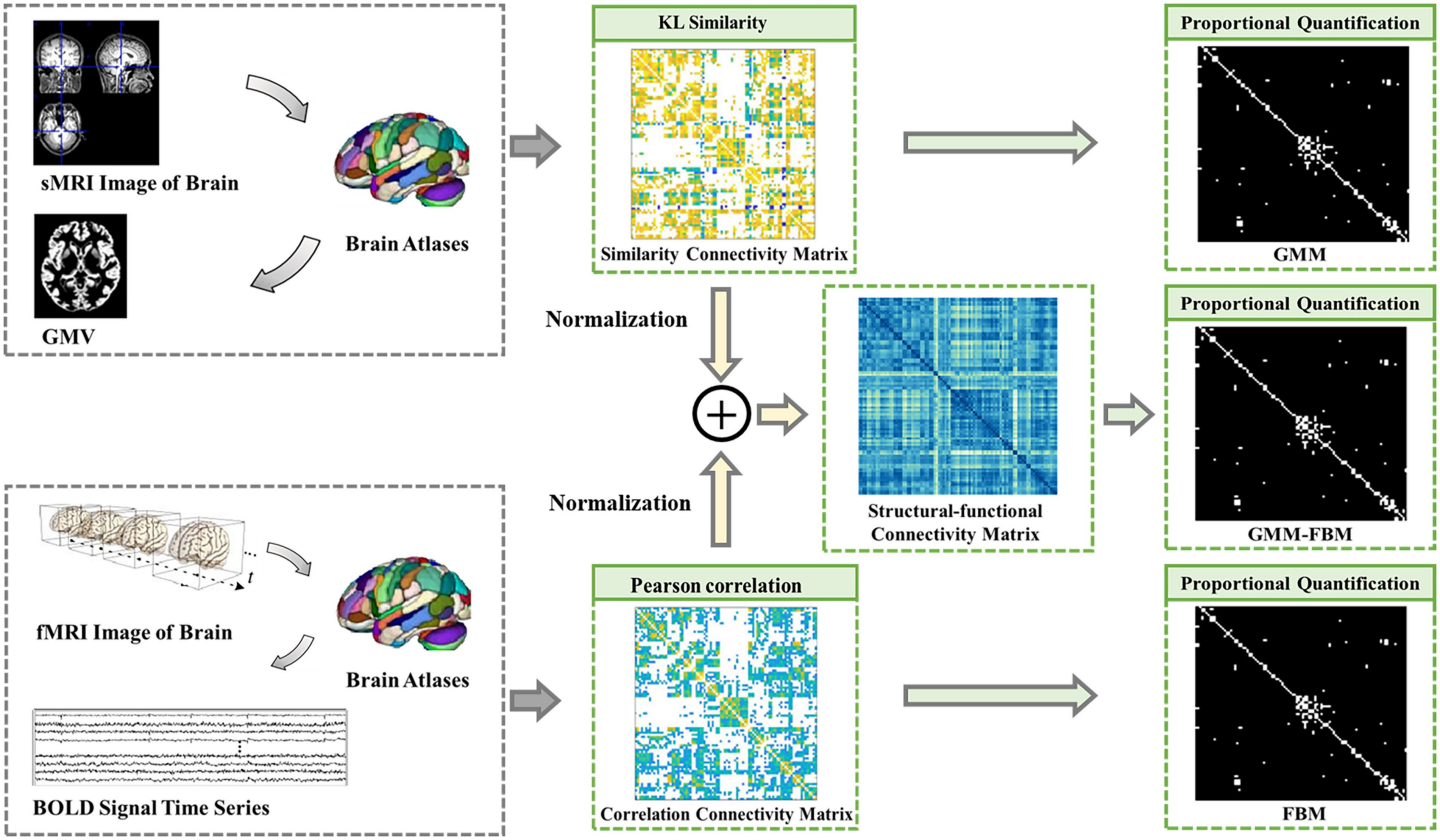
Flow chart of constructing the brain network connection matrices. GMM, Gray Matter Matrix. FBM, Functional Brain Matrix. GMM-FBM, Gray Matter Matrix-Functional Brain Matrix. Similarity connectivity matrix, correlation connectivity and structural-functional connectivity matrix were all the weight fully connected adjacency matrices. The proportional quantification method was proposed to construct the sparse binary adjacency matrices, including GMM, FBM and GMM-FBM.

The brain network can be modeled as a graph consisting of brain regions as the nodes and their connectivity as the edges. The construction methods of the brain network based on MRI data were the same as those in our previous studies (Kong et al., 2021; Wang et al., 2022). In this section, we constructed brain connectivity matrices as edge features, including structural connectivity matrices, functional connectivity matrices and structural-functional connectivity matrices, based on sMRI and fMRI data. The flow chart of constructing the brain connectivity matrices is shown in Figure 1.

The structural brain network constructed from sMRI data was based on the GMV map cut out in the preprocessing process, so the structural connectivity matrix was defined as the gray matter matrix (GMM). The edges of the structural brain network were defined as the Kullback–Leibler (KL) divergence-based similarity (Kong et al., 2014; Wang et al., 2016; Kong et al., 2021) measure between two ROIs of the GMV map. The functional brain network constructed from the fMRI data and the functional connectivity matrix was defined as the functional brain matrix (FBM). The edges of the functional brain network were defined as the absolute Pearson correlation coefficient between the regional mean time series. In addition, the structural-functional connectivity matrix constructed by combining sMRI and fMRI data was defined as the gray matter matrix-functional brain matrix (GMM-FBM). Specifically, the similarity matrix extracted by sMRI and the correlation connectivity matrix extracted by fMRI were normalized separately and then added to obtain the structural-functional connectivity matrix. The GMM, FBM and GMM-FBM obtained based on the AAL atlas were 90 × 90 symmetric adjacency matrices, and those obtained based on the BNA atlas were 246 × 246 symmetric adjacency matrices. According to the calculation process, the initial brain network of each subject was a complete network, where each node was connected with all the other nodes. However, considering all the correlations may incorporate the spurious and weak connections, which are most influenced by experimental noise (Li L. et al., 2021). To screen more important connections, speed up the calculation of the model, and prevent the model from overfitting and oversmoothing (Yao et al., 2021), we proposed a proportional quantification method to construct sparse binary networks. First, we defined the threshold as the percentile value of the edge weights, which was calculated according to the quantification parameter. Second, the edge was retained if its weight was larger than the threshold, and the weight was reset to 1. According to the characteristics of the Erdős-Rényi network, the sparsity of the fully connected network is at least 2lnN/N, where N is the number of nodes (Erdös and Rényi, 1959; Schindler et al., 2008). Therefore, we reserved the top 10 and 5% edges of the brain graph constructed based on the AAL and BNA atlases, respectively. Therefore, we obtained 6 types of adjacency matrices for each subject based on two brain atlases and two modalities of MRI data.

#### 2.3.3. Construction of brain graphs by combining node features and edge features

Each subject was represented as a brain graph that contained a feature matrix and an adjacency matrix, in which the feature matrix was regarded as node features and the adjacency matrix was regarded as edge features. For each brain atlas, there were 3 types of node features, including sMRI features (GMV, WMV and sDC), fMRI features (ReHo, ALFF and fDC) and sMRI + fMRI features (GMV, WMV, sDC, ReHo, ALFF and fDC), and 3 types of edge features, including GMM, FBM and GMM-FBM. Therefore, we can generate 9 types of brain graphs by pairwise combinations of node features and edge features for each brain atlas, which are shown in Table 2.

**Table 2:** Nine types of brain graphs by pairwise combination of edge features and node features for each brain atlas.

Type of brain graph	Edge features	Node features
① GMM & sMRI	GMM	sMRI
② GMM & fMRI	GMM	fMRI
③ GMM & sMRI + fMRI	GMM	sMRI + fMRI
④ FBM & sMRI	FBM	sMRI
⑤ FBM & fMRI	FBM	fMRI
⑥ FBM & sMRI + fMRI	FBM	sMRI + fMRI
⑦ GMM-FBM & sMRI	GMM-FBM	sMRI
⑧ GMM-FBM & fMRI	GMM-FBM	fMRI
⑨ GMM-FBM & sMRI + fMRI	GMM-FBM	sMRI + fMRI

Node features of sMRI including GMV, WMV and sDC. Node features of fMRI including ReHo, ALFF and fDC. Node features of sMRI + fMRI including GMV, WMV, sDC, ReHo, ALFF and fDC.

### 2.4. Graph convolution network algorithm

#### 2.4.1. Architecture of graph convolutional network

**Figure 2:**
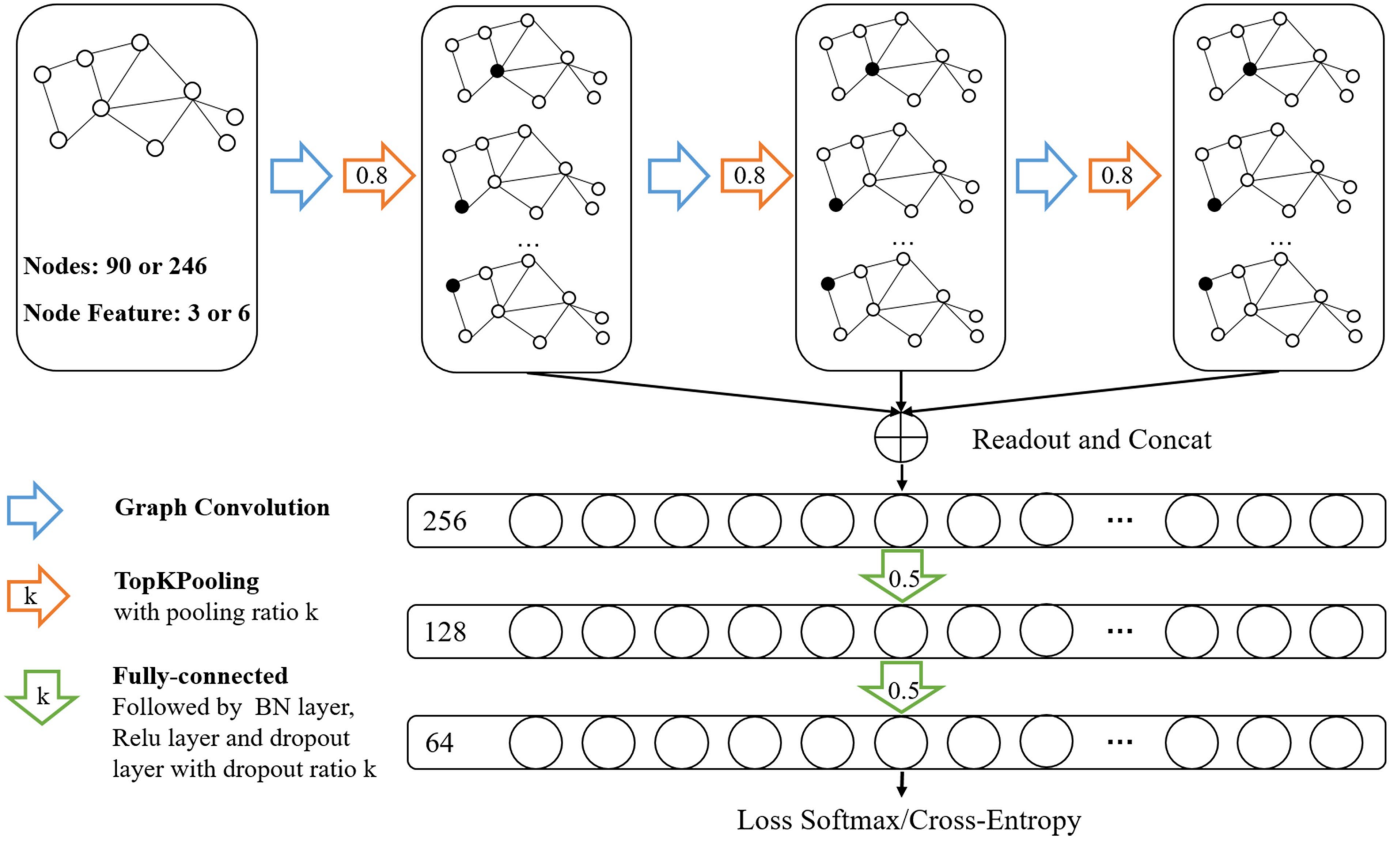
Architecture of Graph Convolutional Networks. Input data was brain graph which consisted of 90 nodes (based on AAL atlas) or 246 nodes (based on BNA atlas), and the number of node features was 3 (sMRI features or fMRI features) or 6 (sMRI + fMRI features). The architecture of GCN comprised three blocks consisting of a graph convolutional layer, a graph pooling layer and a readout layer. The readout layers were implemented by concatenating global max-pooling with global mean-pooling. Node features were aggregated to a fixed size representation in each readout layer and then fed their summation to the fully connected layers for classification.

In this study, we formulated SZ diagnosis as a graph classification task and used the GCN method to discriminate SZ patients based on a combined multimodal MRI and connectomics analysis. Unlike node-level tasks, graph classification tasks need to focus on both the structure information of the graph and the characteristic information of each node. GCN, an extension of CNN in the non-European domain, can simultaneously learn node characteristic information and graph structure information. The architecture of the GCN classifier (Figure 2) were the same with our previous study (Chen et al., 2023) that adopted the GCN framework introduced in Lee et al. (2019). The GCN classifier implemented a hierarchical pooling architecture (Cangea et al., 2018; Lee et al., 2019) and comprised three blocks consisting of a graph convolutional layer, a graph pooling layer and a readout layer. The fully connected layers were used after the aggregation of the readout layers to make classification decisions.

The graph convolution operation is defined as (Kipf and Welling, 2017):


(1) $$H^{\left(l+1\right)}=ReLu\left({\tilde{D}}^{-\frac{1}{2}}\tilde{A}{\tilde{D}}^{-\frac{1}{2}}H^{\left(l\right)}W^{\left(l\right)}\right)$$

where $H^{\left(l+1\right)}\epsilon \;{\mathbb{R}}^{N\times D^{\left(l+1\right)}}$ is the feature matrix of the ${\left(l+1\right)}^{th}$ layer, *N* is the number of nodes and $D^{\left(l+1\right)}$ is the feature dimension of the ${\left(l+1\right)}^{th}$ layer. $H^{\left(l\right)}\epsilon \;{\mathbb{R}}^{N\times D^{\left(l\right)}}$ and $D^{\left(l\right)}$ are the feature matrix and the feature dimension of the $l^{th}$ layer, respectively. $\tilde{A}=A+I_{N}$ is the adjacency matrix with added self-connections, and $I_{N}$ is the identity matrix. $\tilde{D_{ij}}=\underset{j}{\sum }\tilde{A_{ij}}$ and $W^{\left(l\right)}$ are layer-specific trainable weight matrices. ReLu is the activation function.

**Figure 3:**
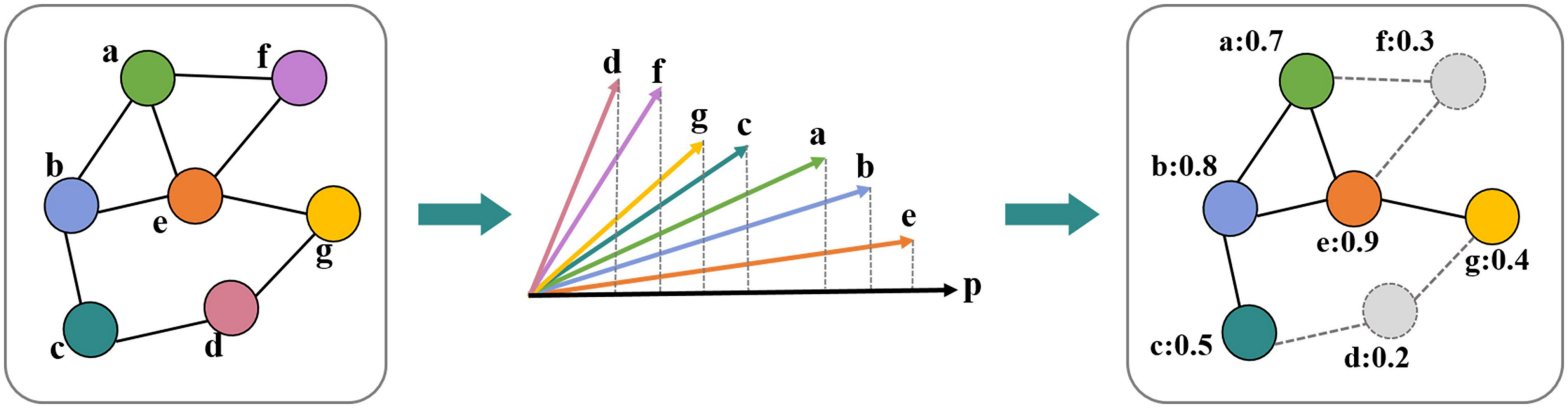
Operation in the TopK pooling layer. Each node feature vector was projected into a scalar value based on a trainable projection vector.

After the convolution, we applied the TopK pooling method to select a portion of nodes to form a coarsened graph. The TopK pooling layer projected each node feature vector into a scalar value used as the ranking score, which considered all features (Cangea et al., 2018; Gao and Ji, 2021). We obtained the scalar projection vector *y* of each node based on a trainable projection vector *p* by matrix multiplication. By using the ranking scores $y^{\left(l\right)}$ of the $l^{th}$ layer, the coarsened graph is formed by computing the new adjacency matrix *A*^(*l* + 1)^ and feature matrix *H*^(*l* + 1)^. The operation of the TopK pooling layer (Figure 3) was as follows:


(2) $$y^{\left(l\right)}=\frac{H^{\left(l\right)}p^{\left(l\right)}}{{\left|\left|p^{\left(l\right)}\right|\right|}_{2}}$$

(3) $$i=top_{k}\left(y^{\left(l\right)}\right)$$

(4) $$H^{\left(l+1\right)}={\left(H^{\left(l\right)}⊙tahn\left(y^{\left(l\right)}\right)\right)}_{i}$$

(5) $$A^{\left(l+1\right)}=A_{i,i}^{\left(l\right)}$$

Here, ||.|| is the *L2* norm. The notation finds the indices corresponding to the largest k elements in score vector y. ⊙ is (broadcasted) element-wise multiplication, and (.)_i,j_ is an indexing operation that takes elements at row indices i and column indices j (no indices j denotes all indices). The *ratio* of the graph pooling layer is the hyperparameter used to compute


k=ratio.N.

The TopK pooling method improved the fusion efficiency of remote nodes by dropping nodes layer by layer, which may lead to a lack of the means to effectively merge information on all nodes. Therefore, a readout layer followed each pooling layer to aggregate multiscale global information of the graph by concatenating global max-pooling with global mean-pooling (Cangea et al., 2018; Xu et al., 2018; Lee et al., 2019). Node features were aggregated to a fixed size representation in each readout layer and then fed their summation to the fully connected layers for classification.

#### 2.4.2. Salient brain regions detection from pooling layer

Recent findings indicated that some ROIs were more indicative of predicting SZ patients than others (Wu et al., 2018; Li et al., 2020). We used the last pooling layer to identify discriminative ROIs contributing significantly to the recognition of SZ. For each subject in the training dataset, we took the first 20 ROIs retained in the last pooling layer and counted the frequency of each node. The significance score for each node was obtained by calculating the ratio of the frequency of the node to the total number of nodes (e.g., 300 training samples, 20 nodes per sample, 6,000 nodes in total).

#### 2.4.3. Experimental setup

In our experiment, we evaluated the effectiveness of our method on 345 subjects that were randomly split into two groups. The training dataset contained 300 subjects and the testing dataset contained 45 subjects. To observe the effects of different modalities of MRI data and different brain atlases on the classification performance, 18 types of brain graphs were constructed as the input data of the GCN model. We utilized the metrics of accuracy, the area under the receiver operating characteristic curve (AUC), sensitivity, specificity, F1-score and precision to quantitatively estimate the performance. Considering that the sample size was not large and different training/testing splits lead to dramatically different rankings of models (Shchur et al., 2019; Flint et al., 2021; Sun et al., 2023), we randomly split training and testing datasets 10 times, repeated the experiments and calculated the averaged performance for each type of brain graph. It is worth emphasizing that in our recent fMRI study (Chen et al., 2023), we randomly split training and testing datasets 200 times to get the average performance and we found that the performance obtained on a single data split could be fragile and misleading, confirming the necessity of a multiple data split evaluation strategies. However, there are many models involved in this multimodal (18 types of brain graphs) study, and it would cause great computational complexity if all models repeated 200 times. In the pretest, we found that the average performance based on 10 randomly dataset splitting was basically the same as the average performance based on 200 randomly dataset splitting. Therefore, in order to reduce the computational complexity without compromising the reliability of the generalizability evaluation, we chose to randomly split training and testing datasets 10 times in this study.

The parameter settings of the training procedure are shown in Table 3. To avoid overfitting, we used the dropout technique, batch normalization (BN) and early stopping strategy. The model was implemented using the PyTorch library and the PyTorch Geometric (PyG) library.

**Table 3:** The parameter settings of the training procedure.

Parameter name	Parameters
Optimizer	Adaptive optimizer (Adam)
Loss function	Cross entropy loss
Activation function	ReLu
Learning rate	0.0001
Batch Size	30
Dropout rate	0.5
Maximum epochs	1,000
Patience of early stopping	500

## 3. Results

### 3.1. Classification results of different brain atlases and different MRI data

Based on the construction method of the brain graph in Section 2.3, 18 types of brain graphs can be obtained by combining node features and edge features, which were used as the input graphs of the GCN classifier and the average performance is shown in Table 4. For both the AAL and BNA atlases, the optimal classification performance was achieved when FBM was used as the edge feature and both structural and functional features of ROIs were used as node features. Specifically, the model with *“AAL: FBM & sMRI + fMRI”* as the input graph obtained an average accuracy of 95.8%, and the AUC, sensitivity, specificity, F1-score and precision reached 96.4, 94.8, 96.2, 94.6 and 94.6%, respectively. The model with *“BNA: FBM & sMRI + fMRI”* as the input graph obtained an average accuracy of 94.2%, which was slightly lower than that of the AAL atlas. For brain graphs with multimodal edge features, the best performance was obtained using functional node features for the AAL atlas, while the best performance was obtained using both structural and functional node features for the BNA atlas. In addition, we compared our research with the existing SZ classification studies, which are shown in Table 5. The GCN model with *“AAL: FBM & sMRI + fMRI”* as the input graph in this study showed the best accuracy.

**Table 4:** Classification results of different brain graphs and the best results are in bold.

Brain Atlas	Input Graph	Performance					
		Accuracy (%)	AUC (%)	Sensitivity (%)	Specificity (%)	F1-score (%)	Precision (%)
AAL	GMM & sMRI	73.3	66.0	47.6	89.1	56.6	81.6
	GMM & fMRI	74.9	68.0	57.5	86.1	62.7	77.4
	GMM & sMRI + fMRI	76.4	72.1	51.5	92.0	62.2	82.2
	FBM & sMRI	71.8	67.0	49.3	86.0	57.3	72.7
	FBM & fMRI	92.4	93.6	87.4	95.1	89.3	92.1
	FBM & sMRI + fMRI	**95.8**	**96.4**	**94.8**	**96.2**	**94.6**	**94.6**
	GMM-FBM & sMRI	72.2	66.7	61.7	78.0	64.7	70.2
	GMM-FBM & fMRI	94.0	96.2	91.7	95.2	92.7	94.2
	GMM-FBM & sMRI + fMRI	91.1	93.0	84.5	95.3	88.3	93.7
BNA	GMM & sMRI	78.2	94.5	77.3	78.0	71.9	79.5
	GMM & fMRI	74.7	71.8	70.5	76.5	68.3	69.6
	GMM & sMRI + fMRI	76.7	73.1	62.0	86.0	67.1	76.6
	FBM & sMRI	75.1	71.7	63.4	82.2	66.4	72.7
	FBM & fMRI	92.9	92.5	89.3	95.1	90.6	92.7
	FBM & sMRI + fMRI	94.2	95.0	94.0	94.4	92.7	92.3
	GMM-FBM & sMRI	74.2	68.9	56.9	86.0	63.5	79.3
	GMM-FBM & fMRI	90.0	93.7	82.2	95.7	86.3	92.8
	GMM-FBM & sMRI + fMRI	93.3	94.9	92.9	93.8	92.1	91.6

**Table 5:** Performance comparison of the proposed method with competing methods.

References	Sample Size	Input features	Classifier	Accuracy
Kim et al. (2020)	SZ = 119, NC = 39	Brain network topology properties	LDA	80.66%
Wang et al. (2022)	SZ = 140, NC = 205	Brain network topology properties	SVM	81.20%
Phang et al. (2020)	SZ = 45, NC = 39	Brain network topology properties	CNN	91.69%
Oh et al. (2022)	SZ = 171, NC = 161	Brain graph	BrainNet-GA CNN	83.13%
Chang et al. (2021)	SZ = 80, NC = 32	Brain graph	GCN	93.33%
Our best	SZ = 140, NC = 205	Brain graph	GCN	95.80%

### 3.2. Classification results of different sparsities

**Figure 4:**
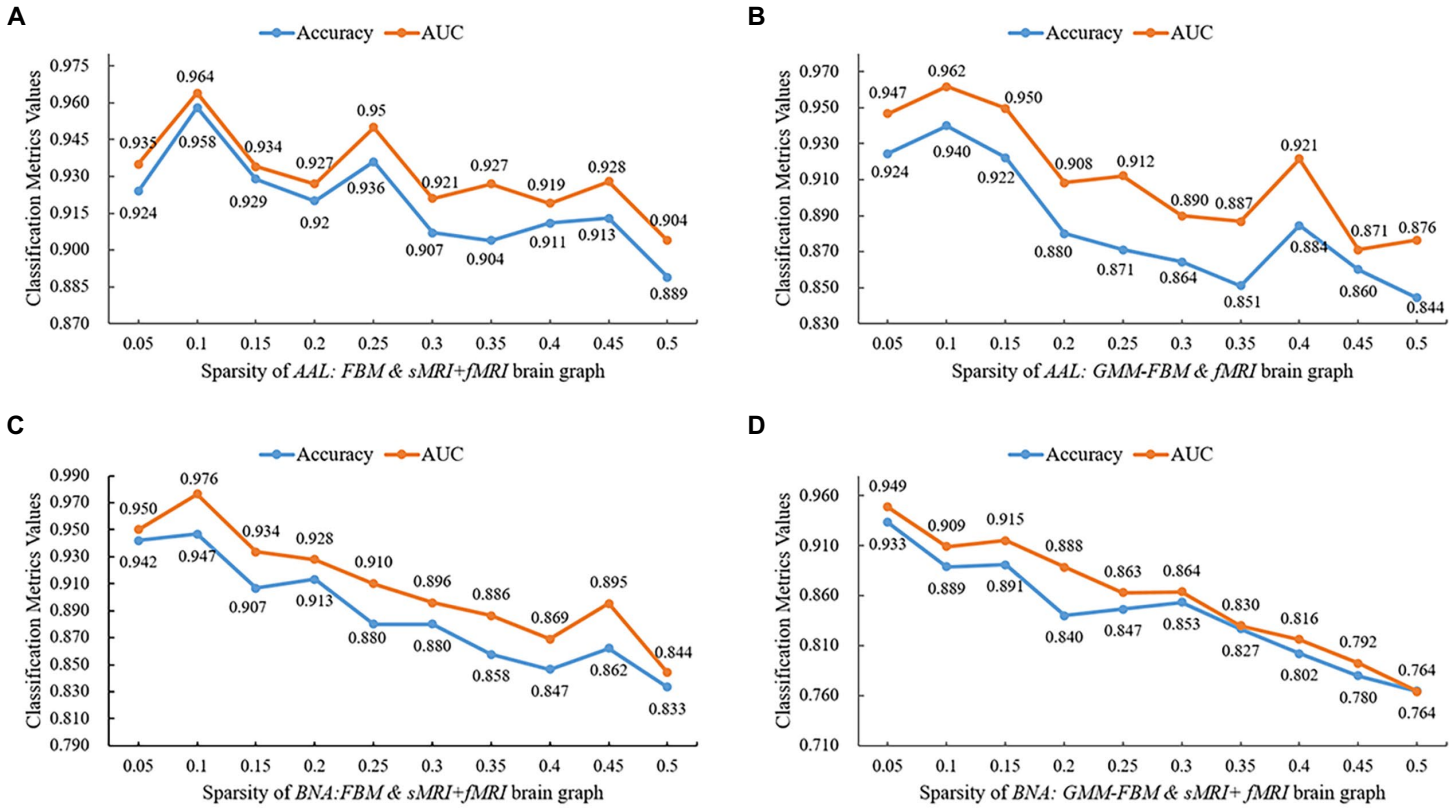
Quantitative performance of brain graphs with different sparsities. **(A)** Results of the *“AAL: FBM & sMRI + fMRI”* models, **(B)** Results of the *“AAL: GMM-FBM & fMRI”* models, **(C)** Results of the *“BNA: FBM & sMRI + fMRI”* models, and **(D)** Results of the *“BNA: GMM-FBM & sMRI + fMRI”* models.

To avoid losing the node-centralized local topology information due to graph convolution on the weighted complete graph, we proposed a proportional quantification method to construct binary brain graphs. Sparse representation-based brain graph construction methods with proportional quantification strategies could generate more robust and biologically meaningful connectivity brain graphs. We defined sparsity as the ratio of the number of preserved edges to the total number of edges. The initial sparsities of brain graphs constructed based on AAL and BNA atlases were set as 10 and 5% separately in Section 3.1. Specifically, the smaller the sparsity, the greater the brain graph focused on reflecting the topological relationships between the more strongly connected brain regions. However, a smaller sparsity means that more weak connections are discarded, which may carry the distinguishing information. In this section, we conducted comparative experiments with different sparsities for the models with single-modal edge feature and multimodal edge feature that achieved better performance in Section 3.1, and two brain atlases were investigated separately. The brain graphs included in this sparsity study were *“AAL: FBM & sMRI + fMRI,” “AAL: GMM-FBM & fMRI,” “BNA: FBM & sMRI + fMRI”* and *“BNA: GMM-FBM & sMRI + fMRI.”* We set the optimization of the sparsity in the range of 5 to 50% with a 5% interval and the results are shown in Figure 4. For the AAL atlas, the optimal values were obtained when sparsity was 10% for both *“AAL: FBM & sMRI + fMRI”* and *“AAL: GMM-FBM & fMRI,”* which were consistent with the initial sparsity. For the BNA atlas, the optimal sparsity of the *“BNA: GMM-FBM & sMRI + fMRI”* model was also consistent with the initial sparsity. However, the *“BNA: FBM & sMRI + fMRI”* model obtained the best performance using a sparsity of 10%. Although 5% was not the optimal sparsity, the *“BNA: FBM & sMRI + fMRI”* model with 5% still obtained superior performance and achieved an accuracy of 94.2%.

In general, the optimal sparsity was small and close to the minimum sparsity of the fully connected network (mentioned in Section 2.3.2). This may be because when combined features are adopted as node features, the combined features will provide more abundant and complementary information, resulting in less reliance of GCN on edge information. Therefore, using a larger proportion that preserved more edges of the brain graph would make the classifier tend to overfitting and thus lead to a decline in classification performance. Meanwhile, the sDC and fDC features were extracted from the edge features, which further increased the redundant information from edge features, leading to overfitting.

### 3.3. The results of salient brain regions

**Figure 5:**
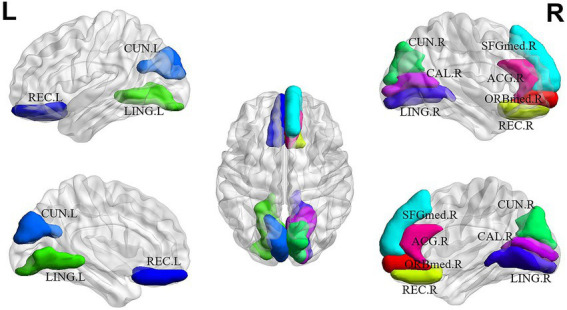
The top 10 salient brain regions based on the *“AAL: FBM & sMRI + fMRI”* brain graph. ORBmed.R: the right medial orbitofrontal cortex; REC.R: the right rectus gyrus; REC.L the left rectus gyrus; LING.L: the left lingual gyrus; CUN.R: the right cuneus; SFGmed.R: the right medial superior frontal gyrus; CUN.L: the left cuneus; LING.R: the right lingual gyrus; CAL.R: the right calcarine cortex; ACG.R: the right anterior cingulate gyrus. The top 10 salient brain regions predominantly distributed in the prefrontal and occipital cortices, were mainly involved in the systems of emotion and visual processing.

For explainability analysis, the TopK graph pooling was used to estimate the contribution of each ROI to GCN classification. We calculated the average significance score across all the training individuals to reflect the contribution of each brain region. Herein, we reported the top 10 salient brain regions of the last pooling layer of the *“AAL: FBM & sMRI + fMRI”* model, and the results are shown in Table 6; Figure 5. The results showed that the top 10 salient brain regions were mostly in the prefrontal cortex and occipital cortex, including the right medial orbitofrontal cortex (ORBmed), the right rectus gyrus (REC), the left REC, the left lingual gyrus (LING), the right cuneus (CUN), the right medial superior frontal gyrus (SFGmed), the left CUN, the right LING, the right calcarine cortex (CAL) and the right anterior cingulate gyrus (ACG). These salient brain regions were mainly involved in emotion and visual processing, which may be related to the clinical symptoms of hallucinations and mood disorders in patients with SZ.

**Table 6:** Top 10 salient brain regions of the last pooling layer of the “*AAL: FBM* *&* *sMRI + fMRI*” model.

No.	Score	Brain region	Cortex
26	0.03171	Orbitofrontal cortex (medial).R (ORBmed.R)	Prefrontal
28	0.03160	Rectus gyrus.R (REC.R)	Prefrontal
27	0.03140	Rectus gyrus.L (REC.L)	Prefrontal
47	0.02836	Lingual gyrus.L (LING.L)	Occipital
46	0.02814	Cuneus.R (CUN.R)	Occipital
24	0.02796	Superior frontal gyrus (medial).R (SFGmed.R)	Prefrontal
45	0.02768	Cuneus.L (CUN.L)	Occipital
48	0.02684	Lingual gyrus.R (LING.R)	Occipital
44	0.02665	Calcarine cortex.R (CAL.R)	Occipital
32	0.02664	Anterior cingulate gyrus.R (ACG.R)	Prefrontal

## 4. Discussion

In this study, we formulated SZ diagnosis as a graph classification problem using a combined multimodal MRI and connectomics analysis. For each brain atlas (AAL and BNA), there were 3 types of node features, including sMRI, fMRI and sMRI + fMRI features, and 3 types of edge features, including GMM, FBM and GMM-FBM. Therefore, we generated 9 types of brain graphs by pairwise combinations of node features and edge features for each brain atlas. Our main findings included the following: (1) the GCN model with *“AAL: FBM & sMRI + fMRI”* as the input graph obtained the highest average accuracy of 95.8%, which outperformed other existing SZ classification studies; (2) in the explainability analysis, the top 10 salient brain regions, predominantly distributed in the prefrontal and occipital cortices, were mainly involved in the systems of emotion and visual processing.

As seen from Table 4, the *“AAL: FBM & sMRI + fMRI”* model achieved the best classification performance with an average accuracy/AUC of 95.8%/96.4%, which was better than the classification performance of the *“AAL: FBM & sMRI”* model and the *“AAL: FBM & fMRI”* model. Similarly, when using GMM as edge features, the model using sMRI + fMRI features as node features achieved an accuracy of 76.4%, which was higher than that of sMRI or fMRI features. In addition, for the BNA atlas, when using the same edge features (FBM, GMM or GMM-FBM), the classification performance with multimodal node features was optimal. These findings suggested that the multimodal node features, which provided richer and complementary information, can improve the classification performance of SZ. Wang et al. (2022) found that the classification performance with multimodal nodal attributes computed by structural and functional brain networks as the input features was significantly better than that of any single-modal nodal attributes, which was consistent with our findings. However, an abnormal finding was that the *“AAL: GMM-FBM & fMRI”* model performed better than the *“AAL: GMM-FBM & sMRI + fMRI”* model. We speculated that the model was less stable due to the insufficient samples and was more sensitive to different training/testing splits. Although the performance of the *“AAL: GMM-FBM & sMRI + fMRI”* model was inconsistent with previously mentioned findings, a high accuracy (91.1%) was obtained.

From the perspective of node features, we found that the fMRI features contributed more to discriminating SZ patients than the sMRI features. One evidence was that when using FBM as the edge features, the accuracy and AUC obtained using fMRI features as node features (accuracy was 92.4%, AUC was 93.6%) were significantly higher than those obtained using sMRI features as node features (accuracy was 71.8%, AUC was 67.0%). The other evidence was that when using GMM as the edge features, the performance achieved using fMRI as node features was better than that achieved using sMRI features as node features, which confirmed our finding and excluded the possibility that using the edge features and node features from the same modal MRI leads to better classification performance. Zang et al. (2021) used combinations of features extracted from three modal MRI images (sMRI, DTI and fMRI) to classify SZ patients and found that the features with the highest ranking contribution to classification were mainly fMRI features, indicating that the fMRI features are more effective and conducive to classification, which was similar to our findings.

From the perspective of edge features, we found that the contribution of the FBM to classification significantly exceeds that of the GMM, and the performance of the GMM-FBM were similar to that of the FBM. As shown in Table 4, when GMM was used as edge features, the classification performance was poor regardless of which node features were selected, and the accuracy was approximately 75.0%. When using the same node features, the classification performance of using FBM or GMM-FBM as the edge features was basically higher than that of using GMM as the edge features.

From the perspective of the brain atlas, we found that the results of the AAL and BNA atlases were basically consistent. The optimal classification result of the BNA atlas was also obtained when using FBM as the edge features and sMRI + fMRI features as node features, which was consistent with the AAL atlas. The highest classification accuracy of 94.2% was slightly lower than that of 95.1% for the AAL atlas.

Table 5 shows that our method achieved the best accuracy compared with the existing SZ classification studies. Compared with traditional machine learning methods such as the LDA algorithm of (Kim et al., 2020) and the SVM algorithm of Wang et al. (2022), the results indicated that the combination of the GCN method and brain network has better classification performance than the combination of the classical classification algorithm and extracting topological properties of the brain network. The significant improvement in classification performance can be attributed to the advantages of the GCN method in the extraction of characteristics. The training process of traditional machine learning mainly relies on the prior understanding of the brain network, which requires manual selection and extraction of characteristics. These characteristics were obviously insufficient in SZ classification. However, the GCN method can automatically extract enough characteristics for classification during the training process by the backpropagation algorithm. In addition, the classification performance of the GCN method was also better than that of classical deep learning methods such as the CNN method used by (Phang et al., 2020). We can simultaneously learn the relationship between different nodes and edges in the structure of the graph data, which helps to explore the complex associations and patterns between brain regions. This high-level relationship is difficult to formulate but can be represented by the nonlinear combinations in the GCN model and contribute to the final classification decision.

In the explainability analysis, we reported that the top 10 salient brain regions, predominantly distributed in the prefrontal and occipital cortices, were mainly involved in the systems of emotion and visual processing. There were 4 salient brain regions from the prefrontal cortices, including the right ORBmed, the right REC, the left REC and the right ACG, which were involved in emotion processing. The medial prefrontal cortex plays an essential role in many brain functions, including cognitive processes, regulation of emotion, motivation and sociability. Lesions of the medial prefrontal cortex, leading to the impairment of these functions, have been implicated in SZ (Xu et al., 2019). Moreover, there were 5 salient brain regions from the occipital cortices, including the left LING, right CUN, left CUN, right LING and right CAL, which are involved in the visual system. The structural and functional abnormalities of the occipital cortices were highly related to visual hallucinations, one of the main symptoms of SZ patients, which was consistent with previous studies (Wu et al., 2018; Keshavan et al., 2020). Additionally, as shown in Figure 5, the salient brain regions were highly symmetrical and spatially coherent, consistent with the previous finding that ROI relevance should be distributed across the brain cortex (Sebenius et al., 2021).

There were three improvements in this study, compared with our previous study (Chen et al., 2023). First, previous studies have indicated that multimodal MRI was more useful than that single-modal MRI data in the discriminative analyses of SZ patients (Wu et al., 2018; Sebenius et al., 2021; Zang et al., 2021; Wang et al., 2022). In this study, we computed nodal and edge features by the analysis of multimodal MRI data. Importantly, we found that combined edge features by functional MRI data with node features by multimodal MRI data (*“AAL: FBM & sMRI + fMRI”* model) achieved the highest accuracy. Second, in addition to the AAL atlas, we also performed the analysis using the BNA atlas and demonstrated that the results using the BNA atlas were consistent with those using the AAL atlas. These findings indicated that the proposed method in this study was robust. Third, we proposed to use the TopK pooling layer to analyze the contribution of features to the classification modal. Our results indicated that the top 10 salient brain regions were mainly involved in the systems of emotion and visual processing.

Several limitations need to be addressed in the present study. First, the sample size was modest. A deep neural network has a strong expression ability due to its complex structure, so more samples are needed to obtain a more stable and reliable model and to avoid overfitting. In this study, we reported more reliable generalization by repeating the experiment 10 times. However, fluctuations in generalization evaluation may still occur due to different training/testing splits, and as mentioned earlier, certain findings were inconsistent with others, and we speculated that it was related to insufficient samples. Second, we used the selected-based TopK pooling method as the explainability technique on GCN and reported the salient brain regions, which did not mean that GCN was no longer a black box. Recently, the explainability of graph neural networks on graph data has experienced rapid developments (Baldassarre and Azizpour, 2019; Pope et al., 2019; Yuan et al., 2022). However, there is neither a unified treatment of GCN explainability methods nor a standard benchmark and testbed for evaluations. In future studies, we could perform a comparison of different explainability approaches on the GCN classifier for SZ.

## 5. Conclusion

In this study, we formulated SZ diagnosis as a graph classification problem and used the GCN method to classify SZ patients based on a combined multimodal MRI and connectomics analysis. The GCN model with *“AAL: FBM & sMRI + fMRI”* as the input graph obtained the highest average accuracy of 95.8%, which outperformed other existing SZ classification studies. In the explainability analysis, we reported the top 10 salient brain regions, predominantly distributed in the prefrontal cortex and occipital cortex that were mainly involved in emotion and visual processing. This study indicated that the GCN method based on a combined multimodal MRI and connectomics analysis was a promising method to improve the classification performance of SZ patients.

## Data availability statement

Due to the nature of this research, participants of this study did not agree for their data to be shared publicly. Requests to access the datasets should be directed to KW, kaiwu@scut.edu.cn.

## Ethics statement

The studies involving human participants were reviewed and approved by The Ethics Committee of the Affiliated Brain Hospital of Guangzhou Medical University. The patients/participants provided their written informed consent to participate in this study.

## Author contributions

XC and PK: conceptualization, methodology, software, investigation, visualization, formal analysis, data curation, and writing – original draft. RP: writing -review & editing and formal analysis. JH, LL, YH, JZ, HL, GM, XL, and YN: writing – review & editing. FW and KW: writing – review & editing and funding acquisition. All authors contributed to the article and approved the submitted version.

## Funding

This work was supported by the National Key Research and Development Program of China (2021YFC2009400 and 2021YFC2009404), the National Natural Science Foundation of China (72174082, 82271953, and 81971585), Guangdong Basic and Applied Basic Research Foundation Outstanding Youth Project (2021B1515020064), the Key Research and Development Program of Guangdong (2018B030335001, 2020B0101130020, and 2020B0404010002), Guangdong Basic and Applied Basic Research Foundation (2019A1515110427), the Science and Technology Program of Guangzhou (201903010032, 202103000032, 202206060005, 202206080005, 202206010077, and 202206010034), and Key Laboratory Program of Guangdong Provincial Education Department (2020KSYS001).

## Conflict of interest

The authors declare that the research was conducted in the absence of any commercial or financial relationships that could be construed as a potential conflict of interest.

## Publisher’s note

All claims expressed in this article are solely those of the authors and do not necessarily represent those of their affiliated organizations, or those of the publisher, the editors and the reviewers. Any product that may be evaluated in this article, or claim that may be made by its manufacturer, is not guaranteed or endorsed by the publisher.
