# Corrigendum: Discriminative analysis of schizophrenia patients using graph convolutional networks: a combined multimodal MRI and connectomics analysis

**DOI:** 10.3389/fnins.2023.1228322

**Published:** 2023-06-06

**Authors:** Xiaoyi Chen, Pengfei Ke, Yuanyuan Huang, Jing Zhou, Hehua Li, Runlin Peng, Jiayuan Huang, Liqin Liang, Guolin Ma, Xiaobo Li, Yuping Ning, Fengchun Wu, Kai Wu

**Affiliations:** ^1^Department of Biomedical Engineering, School of Biomedical Sciences and Engineering, South China University of Technology, Guangzhou International Campus, Guangzhou, China; ^2^Department of Emotional Disorders, The Affiliated Brain Hospital of Guangzhou Medical University, Guangzhou, China; ^3^Guangdong Engineering Technology Research Center for Translational Medicine of Mental Disorders, Guangzhou, China; ^4^School of Material Science and Engineering, South China University of Technology, Guangzhou, China; ^5^National Engineering Research Center for Tissue Restoration and Reconstruction, South China University of Technology, Guangzhou, China; ^6^Guangdong Province Key Laboratory of Biomedical Engineering, South China University of Technology, Guangzhou, China; ^7^Department of Radiology, China-Japan Friendship Hospital, Beijing, China; ^8^Department of Biomedical Engineering, New Jersey Institute of Technology, Newark, NJ, United States; ^9^Department of Psychosomatic, The Affiliated Brain Hospital of Guangzhou Medical University, Guangzhou, China; ^10^Department of Psychiatry, The Affiliated Brain Hospital of Guangzhou Medical University, Guangzhou, China; ^11^Department of Nuclear Medicine and Radiology, Institute of Development, Aging and Cancer, Tohoku University, Sendai, Japan

**Keywords:** schizophrenia, graph convolutional network, discriminative analysis, human brain connectomics, multimodal MRI

**Incorrect Author Name**

In the published article, an author name was incorrectly written as LiQing Liang. The correct spelling is Liqin Liang.

The authors apologize for this error and state that this does not change the scientific conclusions of the article in any way. The original article has been updated.

